# Infection Hospitalization Trends Among US Home Healthcare Patients, 2013–2018

**DOI:** 10.1017/ash.2021.4

**Published:** 2021-07-29

**Authors:** Ashley Chastain, Jordan Harrison, Jingjing Shang, E. Yoko Furuya, Andrew Dick, Mark Sorbero, Patricia Stone

## Abstract

**Background:** Infections are a frequent cause of hospital (re)admissions for older adults receiving home health care (HHC) in the United States. However, previous investigators have likely underestimated the prevalence of infections leading to hospitalization due to limitations of identifying infections using Outcome and Assessment Information Set (OASIS), the standardized assessment tool mandated for all Medicare-certified HHC agencies. By linking OASIS data with inpatient data from the Medicare Provider Analysis and Review (MedPAR) file, we were able to better quantify infection hospitalization trends and subsequent mortality among HHC patients. **Method:** After stratification (by census region, ownership, and urban or rural location) and random sampling, our data set consisted of 2,258,113 Medicare beneficiaries who received HHC services between January 1, 2013, and December 31, 2018, from 1,481 Medicare-certified HHC agencies. The 60-day HHC episodes were identified in OASIS. Hospital transfers reported in OASIS were linked with corresponding MedPAR records. Our outcomes of interest were (1) hospitalization with infection present on admission (POA); (2) hospitalization with infection as the primary cause; and (3) 30-day mortality following hospitalization with infection as the primary cause. We identified bacterial (including suspected) infections based on *International Classification of Disease, Ninth Revision* (ICD-9) and ICD-10 codes in MedPAR. We classified infections by site: respiratory, urinary tract, skin/soft tissue, intravenous catheter-related, and all (including other or unspecified infection site). We also identified sepsis diagnoses. **Result:** From 2013 through 2018, the percentage of 60-day HHC episodes with 1 or more hospital transfers ranged from 15% to 16%. Approximately half of all HHC patients hospitalized had an infection POA. Over the 6 years studied, infection (any type) was the primary cause of hospitalization in more than a quarter of all transfers (25.86%–27.57%). The percentage of hospitalizations due to sepsis increased from 7.51% in 2013 to 11.49% in 2018, whereas the percentage of hospitalizations due to respiratory, urinary tract, or skin/soft-tissue infections decreased (p <0.001). Thirty-day mortality following a transfer due to infection ranged from 14.14% in 2013 to 14.98% in 2018; mortality rates were highest following transfers caused by sepsis (23.14%-26.51%) and respiratory infections (13.07%-14.27%). **Conclusion:** HHC is an important source of post-acute care for those aging in place. Our findings demonstrate that infections are a persistent problem in HHC and are associated with substantial 30-day mortality, particularly following hospitalizations caused by sepsis, emphasizing the importance of infection prevention in HHC. Effective policies to promote best practices for infection prevention and control in the home environment are needed to mitigate infection risk.

**Funding:** No

**Disclosures:** None

